# *Streptococcus pyogenes* Causing Skin and Soft Tissue Infections Are Enriched in the Recently Emerged *emm*89 Clade 3 and Are Not Associated With Abrogation of CovRS

**DOI:** 10.3389/fmicb.2018.02372

**Published:** 2018-10-09

**Authors:** Catarina Pato, José Melo-Cristino, Mario Ramirez, Ana Friães, The Portuguese Group for the Study of Streptococcal Infections, Teresa Vaz

**Affiliations:** Author Affiliations: Centro Hospitalar do Barlavento Algarvio; Centro Hospitalar de Entre Douro e Vouga; Centro Hospitalar de Leiria; Centro Hospitalar de Vila Nova de Gaia/Espinho; Centro Hospitalar do Alto Ave; Centro Hospitalar do Porto; Centro Hospitalar da Póvoa do Varzim/Vila do Conde; Hospital Central do Funchal; Centro Hospitalar de Lisboa Central; Centro Hospitalar Lisboa Norte; Centro Hospitalar Lisboa Ocidental; Centro Hospitalar do Baixo Vouga; Hospital de Vila Real; Hospitais da Universidade de Coimbra; Hospital de Cascais; Hospital de São João, Porto; Hospital de Braga; Hospital de Santa Luzia, Elvas; Hospital dos SAMS, Lisboa; Hospital Dr. Fernando da Fonseca, Amadora/Sintra; Hospital do Espírito Santo, Évora; Hospital Garcia de Orta, Almada; Hospital Pedro Hispano, Matosinhos; Unidade Local de Saúde do Baixo Alentejo, Beja.; Faculdade de Medicina, Instituto de Microbiologia, Instituto de Medicina Molecular, Universidade de Lisboa, Lisboa, Portugal

**Keywords:** *Streptococcus pyogenes*, skin and soft tissue infections, emm89, covRS, SpeB, ropB, emm clusters, antimicrobial resistance

## Abstract

Although skin and soft tissue infections (SSTI) are the most common focal infections associated with invasive disease caused by *Streptococcus pyogenes* (Lancefield Group A streptococci - GAS), there is scarce information on the characteristics of isolates recovered from SSTI in temperate-climate regions. In this study, 320 GAS isolated from SSTI in Portugal were characterized by multiple typing methods and tested for antimicrobial susceptibility and SpeB activity. The *covRS* and *ropB* genes of isolates with no detectable SpeB activity were sequenced. The antimicrobial susceptibility profile was similar to that of previously characterized isolates from invasive infections (iGAS), presenting a decreasing trend in macrolide resistance. However, the clonal composition of SSTI between 2005 and 2009 was significantly different from that of contemporary iGAS. Overall, iGAS were associated with *emm*1 and *emm*3, while SSTI were associated with *emm*89, the dominant *emm* type among SSTI (19%). Within *emm*89, SSTI were only significantly associated with isolates lacking the *hasABC* locus, suggesting that the recently emerged *emm*89 clade 3 may have an increased potential to cause SSTI. Reflecting these associations between *emm* type and disease presentation, there were also differences in the distribution of *emm* clusters, sequence types, and superantigen gene profiles between SSTI and iGAS. According to the predicted ability of each *emm* cluster to interact with host proteins, iGAS were associated with the ability to bind fibrinogen and albumin, whereas SSTI isolates were associated with the ability to bind C4BP, IgA, and IgG. SpeB activity was absent in 79 isolates (25%), in line with the proportion previously observed among iGAS. Null *covS* and *ropB* alleles (predicted to eliminate protein function) were detected in 10 (3%) and 12 (4%) isolates, corresponding to an underrepresentation of mutations impairing CovRS function in SSTI relative to iGAS. Overall, these results indicate that the isolates responsible for SSTI are genetically distinct from those recovered from normally sterile sites, supporting a role for mutations impairing CovRS activity specifically in invasive infection and suggesting that this role relies on a differential regulation of other virulence factors besides SpeB.

## Introduction

*Streptococcus pyogenes* (group A streptococcus, GAS) is responsible for a variety of human infections ranging from mild and frequent diseases, such as pharyngitis and cutaneous infections, to more severe and rare invasive infections including sepsis, necrotizing fasciitis and streptococcal toxic shock syndrome (Walker et al., 2014). In 2005, the estimated global incidence of invasive GAS infections (iGAS) was 663,000 new cases, resulting in 163,000 deaths, while at least 111 million children under 15 years suffered from pyoderma, mostly in developing countries, and pharyngitis incidence was estimated at over 616 million cases (Carapetis et al., 2005). Even though mild infections are usually self-limited, they may play a crucial role in transmission. Furthermore, the nasopharyngeal mucosa and the skin can also be asymptomatically colonized representing primary reservoirs of GAS (Cunningham, 2000).

The gold-standard typing methodology of GAS is *emm* typing, which relies on the variability of the amino acid sequence of the N-terminal portion of *S. pyogenes* major virulence factor: the M protein (McMillan et al., 2013). The sequence of the 5′ variable region of the *emm* gene encoding the M protein determines the *emm* type, of which there are more than 250 distinct variants (https://www.cdc.gov/streplab/m-proteingene-typing.html). However, *emm* typing is based on the sequence of only approximately 10–15% of the complete *emm* gene. Recently a new classification was proposed based on *emm* clusters established by phylogenetic analysis of the entire sequence of the *emm* gene of 175 different *emm* types (Sanderson-Smith et al., 2014). Each cluster contains isolates with closely related M proteins that share binding motifs to host proteins and other structural properties. Since isolates with the same *emm* type encode nearly identical M proteins, the *emm* cluster can be inferred from the *emm* type.

Associations between certain *emm* types and specific disease presentations have been established. Of particular importance is the association of iGAS with a contemporary *emm*1 clone, frequently designated as M1T1, which has persisted for decades as the major cause of invasive disease in most developed countries (O'Loughlin et al., 2007; Aziz and Kotb, 2008; Luca-Harari et al., 2009; Friães et al., 2012). Recently, the emergence of a specific *emm*89 clade (clade 3) that rapidly outcompeted the previously circulating *emm*89 strains was reported in multiple countries and associated with an increase in the prevalence of *emm*89 among GAS infections (Friães et al., 2015a; Turner et al., 2015; Zhu et al., 2015). Isolates from clade 3 are characterized by the absence of the *hasABC* locus encoding the hyaluronic acid capsule of GAS, and by a variant *nga-ifs-slo* locus, similar to the one present in M1T1 strains, which is associated with increased expression of NAD-glycohydrolase (NADase) and streptolysin O (SLO) (Turner et al., 2015; Zhu et al., 2015).

Despite the success of *emm* typing, some studies have suggested it is not enough to identify GAS clones and that it must be complemented with other typing methods such as multilocus sequence typing (MLST), superantigen (SAg) gene profiling (Carriço et al., 2006; Friães et al., 2013a), and, more recently, whole genome sequencing (Carriço et al., 2013). However, variability in key virulence factors and regulators within clones defined by these typing methods may have important consequences for the virulence of a particular isolate.

A mouse model of skin and soft tissue infection (SSTI) showed that mutations in the *covRS* two component system were a key step for the transition from a localized to a systemic infection (Sumby et al., 2006). Consistently, several studies reported *covRS* mutations in isolates recovered from human infections (Engleberg et al., 2001; Hasegawa et al., 2010; Ikebe et al., 2010; Lin et al., 2014; Friães et al., 2015b). In these isolates the downregulation of SpeB expression, a potent extracellular cysteine protease, is thought to be fundamental toward the switch to a hipervirulent phenotype (Aziz et al., 2004; Kansal et al., 2010). Transcription of *speB* is also under direct control of RopB (Carroll and Musser, 2011), and naturally occurring mutations in *ropB* were also shown to impair SpeB production (Hollands et al., 2008; Carroll et al., 2011). We reported previously that mutations resulting in the truncation of CovS, which presumably impaired its function, were significantly overrepresented among iGAS compared to pharyngitis in Portugal. However these were only present in 10% of invasive isolates, not explaining why most isolates caused invasive infections. Additionally, among all studied isolates, which included invasive and pharyngeal isolates, 20% had no detectable SpeB activity but no significant association was detected between the presence or absence of SpeB activity and the type of infection (Friães et al., 2015b).

In order to cause a wide spectrum of disease, GAS has to be able to adapt to different environments in the host and despite decades of research there is still no consensus regarding which molecular or phenotypic properties are responsible for an enhanced invasive potential of certain lineages. Although the nasopharyngeal mucosa is usually considered as the main source of isolates causing iGAS in developed countries (Fiorentino et al., 1997), SSTI are commonly reported as the predominant foci associated with invasive disease (Lamagni et al., 2008). This raises the possibility that strains adapted to infect the skin have an increased ability to invade and survive in deeper tissues. However, there is scarce data about the characteristics of GAS isolates responsible for SSTI, especially in developed, temperate climate regions. Most of the studies from these regions report the characteristics of SSTI isolates together with GAS from other non-invasive sites (mostly pharyngeal swabs) when comparing invasive and non-invasive disease (Descheemaeker et al., 2000; Ekelund et al., 2005; Rivera et al., 2006). A few others specify the molecular characteristics of the SSTI isolates subset, but are limited in the number of isolates or are restricted to short time periods (Kittang et al., 2008; Mijač et al., 2010; Vähäkuopus et al., 2012; Tamayo et al., 2014). In this study we characterized 320 isolates from SSTI recovered in Portugal during 2003–2009 for their susceptibility to a panel of antimicrobials, *emm* type, SAg profile, and MLST. The genes conferring resistance to selected antimicrobials were also investigated. In addition, all 320 isolates were tested for SpeB activity and in those without detectable activity we sequenced the *covRS* and *ropB* genes to document any mutations. The SSTI isolates from 2005 to 2009 presented substantial differences relative to a collection of previously partially characterized iGAS isolates recovered in the same period in Portugal (Friães et al., 2007, 2013b). The prevalence of mutations impairing CovRS function was found to be lower when compared with that previously reported among iGAS isolates in Portugal (Friães et al., 2015b).

## Methods

### Bacterial isolates

For this study, 24 hospital laboratories distributed throughout Portugal were asked to send us, on a voluntary basis, all GAS isolated from SSTI between January 2003 and December 2009. The study was approved by the Institutional Review Board of the Centro Académico de Medicina de Lisboa. These were considered surveillance activities and were exempt from informed consent. All methods were performed in accordance with the relevant guidelines and regulations. The data and isolates were de-identified so that these were irretrievably unlinked to an identifiable person. Participation was low in the first 2 years (*n* = 17 in 2003, *n* = 8 in 2004), but subsequently increased (average *n* = 59/year, range 48–77) (Dataset 1 available at http://dx.doi.org/10.6084/m9.figshare.6736313). Overall, 320 non-duplicate GAS isolates from SSTI were recovered and included in the study: 306 from skin and soft tissue exudates (pus) and 14 from skin and soft tissue biopsies. Identification of isolates was performed by colony morphology, β-hemolysis on blood agar, and the presence of the characteristic Lancefield group A antigen (OXOID, Basingstoke, UK). In addition, 247 non-duplicate iGAS isolates recovered in the same hospitals during 2005–2009, which had been partially characterized previously (Friães et al., 2007, 2013b), were also included (Dataset 2 available at http://dx.doi.org/10.6084/m9.figshare.7016663). Strain SF370 was obtained from Colección Española de Cultivos Tipo (CECT5109). Strains were grown at 37°C in Todd Hewitt broth (THB) (BD, Sparks, MD, USA) or in Tryptone Soy Agar (Oxoid, Basingstoke, UK) supplemented with 5% defibrinated sheep blood.

### Antimicrobial susceptibility testing and genetic determinants

Susceptibility tests were performed by disk diffusion according to the guidelines of the Clinical and Laboratory Standards Institute (CLSI) (Clinical Laboratory Standards Institute, 2017) for penicillin, vancomycin, erythromycin, levofloxacin, tetracycline, chloramphenicol, clindamycin and linezolid (Oxoid, Basingstoke, UK). *E*-test strips (BioMérieux, Marcy l'Etoile, France) were used for MIC determination in cases of intermediate susceptibility and to confirm resistance when ≤5 isolates were resistant to a particular antimicrobial. Determination of macrolide resistant phenotype was performed as previously described (Melo-Cristino and Fernandes, 1999). A multiplex PCR reaction for *erm*(B), *erm*(A), and *mef* genes was used on macrolide resistant isolates to identify the resistance conferring genes (Figueira-Coelho et al., 2004). The *mef* positive isolates were further analyzed in order to distinguish between *mef* (A) and *mef* (E) (Silva-Costa et al., 2008). The tetracycline resistance genotype was determined for resistant isolates by a multiplex PCR for the genes *tet*(K), *tet*(L), *tet*(M) and *tet*(O) (Trzcinski et al., 2000).

### Molecular typing

The *emm* typing was performed according to the protocols and recommendations of the Center for Disease Control and Prevention (CDC) (https://www.cdc.gov/streplab/protocol-emm-type.html).

The presence of 11 SAg genes (*speA, speC, speG, speH, speI, speJ, speK, speL, speM, smeZ*, and *ssa*) was tested by two multiplex PCR reactions using the amplification of *speB* and *speF* gene fragments as positive controls (Friães et al., 2013a). Absence of a PCR product for *speB* and *speF* was confirmed by Southern blotting as previously described (Friães et al., 2013a).

MLST analysis was performed for all SSTI isolates (*n* = 320), as well as for the iGAS isolates that had not been previously typed by MLST (*n* = 135/247) (Friães et al., 2007, 2012, 2013b). Allele and sequence type (ST) identification was done using the *S. pyogenes* MLST database (https://pubmlst.org/spyogenes/).

The sequence of the *covRS* and *ropB* loci of all SSTI isolates that presented no proteolytic activity (see below) was determined as previously described (Friães et al., 2015b). For each isolate, the sequences were assembled and compared with the corresponding regions of the genome of strain SF370 (GenBank AE004092), considered as the reference wild-type alleles. Isolates were considered to carry null alleles if the changes found were predicted to result in absence of a functional protein due to nonsense mutations or frameshifts. All new *covRS* and *ropB* sequences identified in this study were deposited in GenBank (accession numbers MH537795-MH537849).

### Determination of proteolytic activity and SpeB expression

All isolates were screened for detectable SpeB activity using a plate assay, as previously described (Friães et al., 2015b). Briefly, single GAS colonies were stab-inoculated into fresh plates of medium containing 0.5-strength Columbia broth, 3% w/v skim milk (BD, Sparks, MD, USA), and 1% w/v agar (Oxoid, Basingstoke, UK). A strain was considered to show proteolytic activity when it presented a translucent zone of size similar to the one of strain SF370 after 24h incubation at 37°C, in three independent assays. On the contrary, a strain was considered not to show proteolytic activity if it did not produce a translucent halo in any of the three assays. For strains in which the results of the three assays were inconclusive or not consistent, detection of SpeB by Western blot was performed (Friães et al., 2015b) and this was considered the final result.

### Statistical analysis

Simpson's index of Diversity (SID) with respective 95% confidence intervals (CI_95%_) was used for the analysis of the typing methodologies and to evaluate the allelic diversity of the *covR, covS*, and *ropB* genes found in this study (Carriço et al., 2006). Two-tailed Fisher's exact test and odds ratios were used to identify significant pairwise associations. Overall differences in the distribution of typing characteristics between infection types was evaluated by the χ^2^-test. The Cochran-Armitage test was used to evaluate trends. Only characteristics grouping ≥10 isolates were considered in statistical tests, with all other isolates grouped together in a single group. The *p*-values for multiple tests were corrected using the false-discovery rate (FDR) linear procedure (Benjamini and Hochberg, 1995). A *p*-value < 0.05 was considered significant for all tests.

## Results

### Antimicrobial susceptibility and genotypic determinants of resistance

All 320 SSTI isolates analyzed (Dataset 1) were susceptible to penicillin, vancomycin, and linezolid. Two isolates were non-susceptible to levofloxacin (MIC = 6 mg/mL and 3 mg/mL, both *emm*89). One was resistant to chloramphenicol (*emm*147).

Macrolide resistance was detected in 33 SSTI isolates (10%) of 12 different *emm* types (Dataset 1), with a significant decreasing trend during the years of the study (*p* < 0.001) (Supplementary Figure 1). The majority of these isolates presented the cMLS_B_ phenotype (constitutive resistance to both erythromycin and clindamycin) and carried the *erm*(B) gene (*n* = 22). The remaining macrolide resistant isolates (*n* = 11) exhibited the M phenotype (resistance to erythromycin and susceptibility to clindamycin) and carried the *mef* (A) gene, except for one isolate that was positive for the *mef* (E) gene.

Tetracycline resistance was detected in 47 SSTI isolates (15%) comprising 25 *emm* types (Dataset 1) with no significant temporal trend (Supplementary Figure 1). The majority of these isolates (*n* = 42) presented only the *tet*(M) gene, while three isolates carried both *tet*(M) and *tet*(L) and two isolates presented solely the *tet*(O) gene. Resistance to tetracycline and macrolides was simultaneously detected in 12 isolates, of which 9 presented the cMLS_B_ phenotype.

### *emm* typing

**Figure 1:**
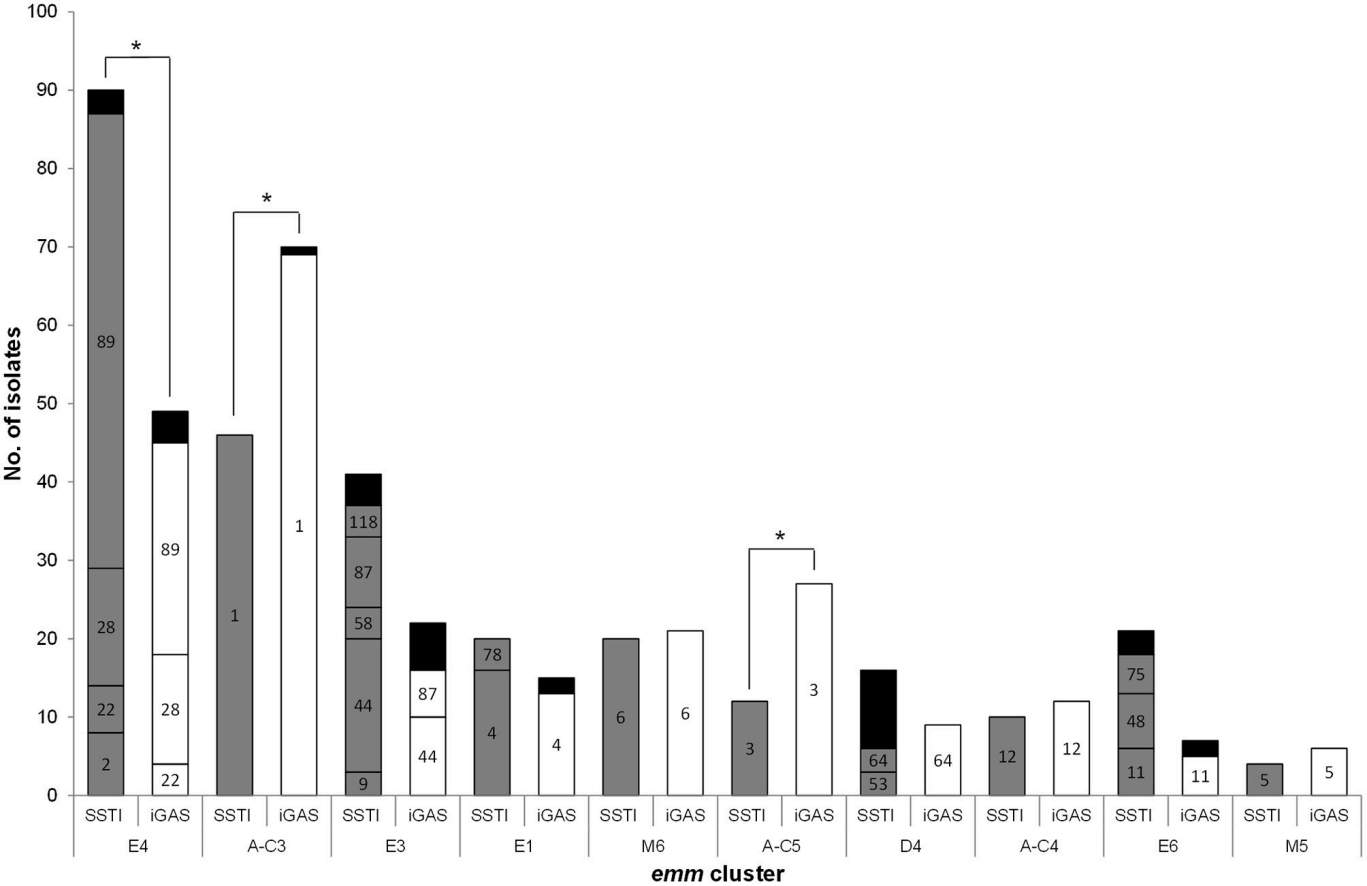
Distribution of isolates recovered from skin and soft tissue infections (SSTI) and invasive infections (iGAS) according to *emm* cluster and *emm* type. Only *emm* clusters with a total of ≥10 isolates in both infection types are represented. Numbers inside the bars represent the *emm* types included in each cluster. Black bars include *emm* types with < 3 isolates [SSTI, E4: *emm*77, *emm*102, and *emm*109 (each *n* = 1); E3, *emm*82, *emm*103, *emm*168, and *emm*209 (each *n* = 1); D4, *emm*70 and *emm*223 (each *n* = 2), *emm*33, *emm*43, *emm*80, *emm*83, *emm*93, and *emm*225 (each *n* = 1); E6:,*emm*65, *emm*81, and *emm*94 (each *n* = 1). iGAS, E4, *emm*77 (*n* = 2), *emm*2, and *emm*84 (each *n* = 1); A-C3: *emm*227 (*n* = 1); E3,*emm*58 and *emm*103 (each *n* = 2), *emm*9 and *emm*118 (each *n* = 1); E1, *emm*78 (*n* = 2); E6, *emm*75 and *emm*81 (each *n* = 1)]. ^*^p ≤ 0.01.

A total of 51 different *emm* types were identified among SSTI isolates (Table 1). Isolates with *emm*89 were the most frequently recovered (*n* = 62, 19%), followed by *emm*1 (*n* = 55, 17%), together accounting for 36% of all SSTI isolates (Figure 1).

**Table 1:** Simpson's Index of Diversity (SID) and respective 95% confidence intervals for *emm* type, *emm* cluster, superantigen (SAg) profile, and sequence type (ST) among skin and soft tissue infections (SSTI) and invasive infections (iGAS) in Portugal.

	**SID (CI**_**95%**_**) [No. partitions]**		
	**SSTI**		**iGAS**
	**2003–2009 (*n* = 320)**	**2005–2009 (*n* = 295)**	**2005–2009 (*n* = 247)**
*emm* type	0.916 (0.899–0.933) [51]	0.919 (0.902–0.936) [49]	0.881 (0.855–0.907) [32]
*emm* cluster ^a^	0.838 (0.813–0.862) [15]	0.842 (0.817–0.867) [15]	0.844 (0.819–0.869) [14]
SAg profile	0.934 (0.922–0.946) [50]	0.935 (0.922–0.948) [49]	0.901 (0.879–0.923) [33]
ST	0.945 (0.933–0.958) [70]	0.949 (0.937–0.961) [68]	0.903 (0.876–0.930) [44]

a *Isolates with no emm cluster (SSTI, n = 3, emm types 127, 147 and 167; iGAS, n = 3, emm types 196, 199, and stG1750) were not considered for SID determination*.

The *emm* types were distributed into 15 *emm* clusters (Table 1 and Figure 1). The majority of SSTI isolates belong to *emm* clusters from clade X (*n* = 189, 59%).

The characteristics of SSTI isolates were compared with those from contemporary iGAS isolates. Given the reduced number of SSTI isolates in the first 2 years of the study, this comparison was performed only for the SSTI and iGAS isolates recovered from 2005 to 2009 (Dataset 1 and Dataset 2). Significant differences were detected in the overall distribution of *emm* types (*p* < 0.001), as well as in the prevalence of specific *emm* types (Supplementary Figure 2) with the SSTI subset presenting a higher SID than the iGAS subset (Table 1, *p* = 0.014). In agreement, 24 *emm* types accounting for 36 isolates were identified exclusively among SSTI, while seven *emm* types accounting for seven isolates were found only in iGAS. While *emm*1 and *emm*3 were significantly overrepresented among iGAS (*p* < 0.001 and *p* = 0.002, respectively), *emm*89 was significantly overrepresented among SSTI (*p* = 0.006). However, when stratifying the *emm*89 isolates as to the presence or absence of the *hasABC* locus (Friães et al., 2015a) (Dataset 1), only the isolates lacking the locus were associated with SSTI (*p* = 0.028). All these differences were still significant after FDR correction. When exploring possible changes in time from 2005 to 2009, there was a significant increase of *emm*89 among SSTI (*p* = 0.012), while among iGAS the increase of *emm*89 was not statistically supported after FDR (Dataset 1 and Dataset 2). In both infection types, this was underpinned by an increase in the proportion of the acapsular *emm*89 isolates (*p* < 0.001), while there was no significant change in time of *emm*89 isolates with the *hasABC* locus in either infection type.

The differences of *emm* types were reflected in the distribution of *emm* clusters among SSTI and iGAS isolates (Figure 1), although the SIDs were not significantly different (Table 1, *p* = 0.910) While iGAS isolates were significantly associated with *emm* clusters A-C3 (comprising almost exclusively *emm*1 isolates) and A-C5 (comprising exclusively *emm*3 isolates) (*p* < 0.001 and *p* = 0.002, respectively), SSTI isolates were significantly associated with *emm* cluster E4 (dominated by *emm*89) (*p* = 0.006).

The *emm* cluster typing system can be used for predicting the ability of the strains to bind different host proteins based on the binding properties of the respective M proteins (Supplementary Table 1) (Sanderson-Smith et al., 2014). When comparing SSTI and iGAS isolates, and excluding the clusters with uncertain binding for each host protein, the ability to bind C4BP was associated with SSTI isolates (*p* < 0.001). This association reflects the high prevalence of clusters E3 and E6 among SSTI, even though the dominant cluster among SSTI (cluster E4) was classified as uncertain in the ability to bind C4BP. SSTI isolates were also associated with the ability to bind IgA and IgG (*p* = 0.002 and *p* = 0.033, respectively), whereas the invasive isolates were associated with the ability to bind fibrinogen and albumin (*p* < 0.001 and *p* = 0.004, respectively), all significant after FDR.

### MLST

The studied SSTI isolates comprised 70 different STs (Tables 1, 2). A total of 20 new STs were found among the SSTI and iGAS isolates typed in this study (ST817-ST836), including two new alleles for *gtr* (108; 109), three for *murI* (105-107), two for *mutS* (88; 89), two for *recP* (125;126), two for *xpt* (102; 103), and three for *yqil* (119-121).

**Table 2:** Distribution of sequence types (ST) among *emm* types identified in skin and soft tissue infections (SSTI) and invasive infections (iGAS) in Portugal.

***emm* type**	**ST (no. of isolates)**	
	**SSTI (2005–2009)^a^ (*n* = 295)**	**iGAS (2005–2009) (*n* = 247)**
1	28 (43); 643 (1); 830 (2)	28 (65); 618 (1); 643 (3)
89	101 (35); 408 (13); 824 (10)	101 (18); 408 (6); 824 (3)
6	382 (13); 411 (7)	382 (13); 411 (8)
3	15 (7); 315 (5)	15 (18); 315 (4); 406 (5)
4	38 (1); 39 (15)	39 (10); 771 (1); 823 (2)
28	52 (15)	52 (12); 458 (1); 821 (1)
44	25 (12); 178 (2); 429 (1); 555 (2)	25 (7); 178 (1); 555 (2)
12	36 (9); 467 (1)	36 (12)
87	62 (9)	62 (6)
64	164 (3)	164 (9)
11	403 (6)	403 (4); 562 (1)
5	99 (4)	99 (6)
22	46 (5); 389 (1)	46 (4)
Others^b^	2 (1); 3 (2); 5 (1);10 (1); 24 (2); 50 (2); 55 (8); 60 (1); 63 (1); 75 (3); 89 (1);120 (1); 130 (3); 150 (3); 161 (7); 164 (1);166 (1); 167 (1); 184 (1); 200 (1); 253 (3); 340 (2); 341 (1); 409 (1); 565 (2); 569 (1); 573 (1); 642 (2); 701 (1); 718 (1); 754 (1); 819 (1); 820 (1); 822 (1); 825 (1); 826 (3); 827 (1); 828 (1); 829 (1); 831 (1); 833 (1); 834 (1); 835 (1); 836 (1)	28 (2); 50 (1); 55 (1); 63 (2); 75 (1); 99 (1); 150 (1); 184 (1); 201 (2); 258 (1); 402 (1); 409 (2); 410 (2); 619 (1); 769 (1); 816 (1); 818 (1), 832 (1); 833 (1)

a *Does not include SSTI isolates recovered during 2003 and 2004 (n = 25), which comprise 13 different STs: [emm1, ST28 (n = 9); emm3, ST15 (n = 1); emm4, ST38 (n = 1); emm12, ST36 (n = 1); emm18, ST642 (n = 1); emm22, ST46 (n = 1); emm28, ST52 (n = 2); emm44, ST25 (n = 1); emm68, ST331 (n = 1); emm73, ST331 (n = 1); emm77, ST63 (n = 1); emm89, ST101 (n = 2) and ST408 (n = 2); emm209, ST817 (n = 1)]*.

b *“Others” include emm types with n < 10 isolates considering both SSTI (2005-2009) and iGAS isolates (2005-2009). Those include emm types 2, 9, 18, 19, 33, 43, 48, 50, 53, 58, 65, 70, 71, 74, 75, 76, 77, 78, 80, 81, 82, 83, 84, 90, 93, 94, 102, 103, 109, 118, 122, 127, 147, 167, 179, 196, 209, 223, 225, 277, and stG1750*.

In the period of 2005–2009, the overall distribution of STs differed significantly between SSTI and iGAS (*p* < 0.001), with a higher diversity among SSTI (Table 1, *p* = 0.002). ST28 and ST15 were associated with iGAS (*p* < 0.001 and *p* = 0.007, respectively) (Supplementary Figure 3), in agreement with the association of the corresponding dominant *emm* types with iGAS (*emm*1 for ST28 and *emm*3 for ST15). These differences were supported after FDR correction.

No significant differences were detected when comparing the ST diversity of isolates with the same *emm* type recovered from each type of infection (Table 2).

### SAg profiling

Overall, chromosomally encoded *smeZ* and *speG* genes were the most frequently detected among SSTI isolates, being present in 307 (96%) and 301 (94%) isolates, respectively (Table 3). PCR-amplification of *speB* and *speF* was not possible for one isolate, and the absence of both genes was confirmed by Southern blot (data not shown). A total of 50 distinct SAg profiles were identified among the SSTI isolates (Tables 1, 3).

**Table 3:** Superantigen (SAg) profiles identified in isolates recovered from skin and soft tissue infections (SSTI) in Portugal during 2003–2009.

**SAg profile^a^**	***speA***	***speC***	***speG***	***speH***	***speI***	***speJ***	***speK***	***speL***	***speM***	***ssa***	***smeZ***	***emm* type (No. of isolates)**
2	+	+	+	–	–	–	+	–	–	–	+	6 (17); 74 (1)
3	+	+	+	–	–	+	–	–	–	–	+	1 (11); 87 (1)
4	+	+	+	–	–	–	–	+	+	–	+	18 (3); 22 (1)
5	+	+	+	–	–	–	–	–	–	–	+	5 (2); 11 (1); 18 (1); 122 (1)
8	+	–	+	–	–	–	+	–	–	+	+	3 (11)
9	+	–	+	–	–	–	+	–	–	–	+	6 (1)
10	+	–	+	–	–	+	–	–	–	–	+	1 (43); 71 (1)
11	+	–	+	–	–	–	–	–	–	–	+	43 (1); 82 (1)
12	–	+	+	+	+	+	–	–	–	+	+	44 (2)
13	–	+	+	+	+	–	–	–	–	+	+	22 (1)
15	–	+	+	+	–	+	+	–	–	–	+	28 (1)
16	–	+	+	+	+	–	–	–	–	–	+	11 (4); 16 (6); 48 (4)
18	–	+	+	–	–	+	+	–	–	+	+	87 (1)
19	–	+	+	–	–	–	+	–	–	+	+	22 (2)
20	–	+	+	–	–	+	–	–	–	+	+	84 (4)
21	–	+	+	–	–	–	–	–	–	+	+	22 (2)
23	–	+	–	–	–	–	–	–	–	+	+	4 (16)
24	–	+	+	–	–	+	+	–	–	–	+	28 (8)
26	–	+	+	–	–	–	+	–	–	–	+	89 (3)
27	–	+	+	–	–	+	–	–	–	–	+	28 (8); 68 (1); 70 (2); 89 (15); 90 (1)
28	–	+	+	–	–	–	–	+	+	–	+	75 (1)
29	–	+	+	–	–	–	–	–	–	–	+	5 (2); 11 (1); 48 (3); 50 (1); 77 (1); 78 (4); 89 (35); 118 (1); 127 (1)
31	–	+	+	–	–	–	–	+	+	–	–	2 (7)
32	–	–	+	+	+	+	–	–	–	+	+	44 (12)
33	–	–	+	+	+	–	–	–	–	–	+	12 (5); 73 (1); 76 (2)
35	–	–	+	+	–	–	–	–	–	–	+	75 (1); 94 (1)
38	–	–	+	–	–	+	–	–	–	+	+	44 (1); 87 (1); 223 (1)
39	–	–	+	–	–	–	–	+	+	+	+	53 (1); 75 (2)
40	–	–	+	–	–	–	–	–	–	+	+	90 (3); 22 (1)
41	–	–	–	–	–	–	–	–	–	+	+	4 (1)
43	–	–	+	–	–	–	+	–	–	–	+	65 (1); 209 (1)
44	–	–	+	–	–	+	–	–	–	–	+	102 (1); 103 (1); 118 (2); 223 (1)
45	–	–	+	–	–	–	–	+	+	–	+	80 (1)
46	–	–	+	–	–	–	–	–	–	–	+	53 (2); 58 (1); 64 (3); 75 (1); 89 (9); 118 (1); 147 (1); 167 (1); 168 (1)
47	–	–	–	–	–	–	–	–	–	–	+	77 (1)
48	–	–	+	–	–	–	–	+	+	–	–	2 (1)
51	+	+	+	+	+	–	+	–	–	–	+	6 (2)
52	+	+	+	–	–	–	+	–	–	+	+	22 (1)
53	+	–	+	–	–	–	–	–	–	+	+	3 (2)
54	–	+	+	+	+	+	–	–	–	–	+	87 (1)
56	–	–	+	+	–	+	–	–	–	–	–	44 (3)
60	+	–	+	+	+	+	–	–	–	–	+	71 (2)
61	+	–	+	–	–	+	+	–	–	–	+	1 (1)
64	–	+	+	+	+	–	–	+	+	–	+	93 (1)
66	–	+	+	–	–	–	–	–	+	–	+	58 (3)
67	–	–	+	+	–	+	–	–	–	+	+	33 (1)
68	–	–	+	+	+	–	–	–	–	–	–	209 (1)
69	–	–	+	+	–	–	–	–	–	–	–	81 (1)
70	–	–	+	–	–	+	+	–	–	–	+	87 (1)
71	–	+	+	–	–	+	–	+	+	–	+	83 (1)

a *The numbering of the SAg profiles follows the one adopted previously (Friães et al., 2013a,b; Silva-Costa et al., 2014)*.

During 2005–2009, the presence of the genes *speA* and *speK* was significantly associated with invasive isolates (*p* < 0.001 and *p* = 0.018, respectively), while *speC, speL*, and *speM* were significantly associated with isolates from SSTI (*p* = 0.019, *p* = 0.003, and *p* < 0.001, respectively) (Supplementary Figure 4A).The overall distribution of SAg profiles differed between the two types of infection (*p* < 0.001) and the SAg profiles of SSTI isolates were more diverse than those of invasive isolates (Table 1, *p* = 0.008). Reflecting the association between *emm* type and infection and the high correlation between *emm* typing and SAg profiling (Friães et al., 2013a), SAg profiles 8 and 10, which correspond mostly to *emm*3 and *emm*1, respectively, were associated with iGAS (*p* = 0.001 and *p* < 0.001, respectively), (Supplementary Figure 4B).

### SpeB protease activity and sequence of *cov*RS and *rop*B

We previously showed that the total proteolytic activity could be used as a proxy for SpeB activity and that all isolates with null alleles, either in *covS* or in *ropB*, lacked SpeB activity (Friães et al., 2015b).

Among the 320 SSTI isolates, 79 (25%) had no detectable SpeB activity (Dataset 1). These isolates represented 22 different *emm* types [SID (CI_95%_) = 0.895 (0.853–0.937)] and 28 STs [SID (CI_95%_) = 0.934 (0.907–0.961)].

**Figure 2:**
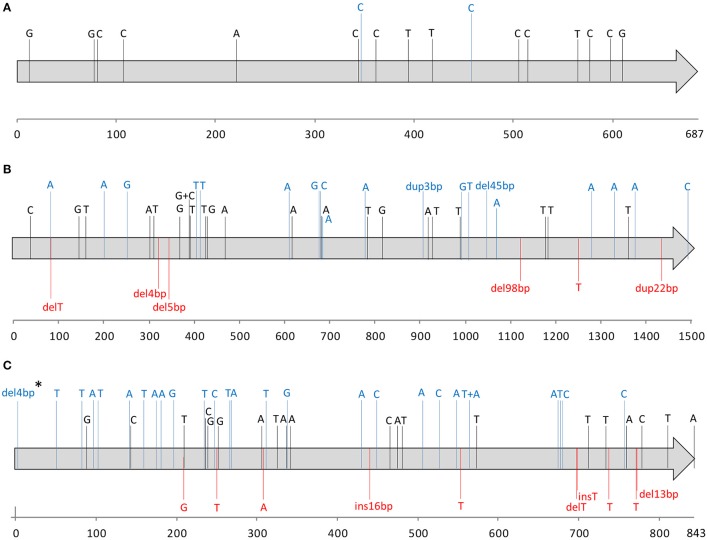
Nucleotide alterations identified in the *covR* **(A)**, *covS* **(B)**, and *ropB* **(C)** alleles among skin and soft tissue isolates with no detectable SpeB (*n* = 79), relative to the alleles present in strain SF370 (AE004092). Each gene is indicated by a gray arrow with the respective numbering scale below. Each sequence variation is indicated in the respective nucleotide position by a letter corresponding to the variant nucleotide, or the sequence or number of base-pairs inserted (ins), deleted (del), or duplicated (dup). Synonymous changes are represented in black; missense mutations and in-frame indels are represented in blue. Changes predicted to result in null alleles, including nonsense mutations and indels that generate frameshifts are represented in red. One exception is represented by “^*^” corresponding to an isolate with an early deletion of 4 bp in *ropB* which is more probable to result in the alteration of the start codon rather than a premature stop codon and therefore was not considered as a null mutation.

Among the isolates with no SpeB activity, 15 distinct *covR* alleles were identified, resulting in only two different amino acid sequences, while 34 and 47 different alleles were detected for *covS* and *ropB*, corresponding to 22 and 37 distinct amino acid sequences, respectively (Figure 2). In two isolates no PCR product was amplified using the *ropB* specific primers, indicating a possible deletion involving the *ropB* gene. In one of these isolates, the absence of PCR amplification of the *speB* and *speF* genes, both located in the same region as *ropB*, supports the occurrence of a large deletion encompassing the entire *ropB* gene, as well as *speB* and *speF*. Deletions spanning different lengths in this locus have been previously identified in GAS isolates from human infections (Friães et al., 2013a).

Nucleotide changes predicted to prevent the expression of a functional protein due to nonsense mutations or frameshifts were considered to result in null alleles. No null alleles were detected in *covR*, while null *covS* alleles were detected in 10 isolates. Since none of the SpeB-positive isolates is expected to have null *covS* alleles, these would correspond to 3% of all isolates recovered from SSTI. These null *covS* alleles were present in isolates of six different *emm* types (*emm*1, *emm*11, *emm*89, and *emm*118, each *n* = 2; *emm*9 and *emm*223, each *n* = 1) and seven STs (ST28, ST403, and ST565, each *n* = 2; ST75, ST101, ST408, and ST828, each *n* = 1), and there were no associations with specific *emm* types or STs. Two isolates presented in-frame indels (45 bp deletion and 3 bp insertion), whose effect on protein function is not clear. The consequences of these in-frame indels in CovS protein function could not be inferred based on the absence of SpeB, since both isolates also carry null *ropB* alleles which could explain the downregulation of SpeB. Therefore these were not considered null alleles.

A total of 12 isolates presented null *ropB* alleles (including the two isolates with a *ropB* deletion), representing 4% of all isolates, and 15% of the isolates with no detectable SpeB. These isolates comprised eight *emm* types (*emm*1, *n* = 3; *emm*12 and *emm*89, each *n* = 2; *emm*2, *emm*3, *emm*11, *emm*22, and *emm*87, each *n* = 1) and nine STs (ST28, *n* = 3; ST36, *n* = 2; ST46, ST55, ST62, ST101, ST315, ST403, and ST408, each *n* = 1), with no association to any specific *emm* type or ST.

Among the isolates without SpeB, a total of 22 (28%) presented a null allele in at least one of the three genes. Forty-four SpeB-negative SSTI isolates carry amino acid changes in *covRS* or *ropB* whose impact on SpeB expression is difficult to predict (Olsen et al., 2012; Friães et al., 2015b; Horstmann et al., 2015). The remaining 13 isolates with no detectable SpeB showed the same amino acid sequence as the reference strain for all sequenced genes (*emm*71, *n* = 1; *emm*80, *n* = 1; *emm*87, *n* = 1; *emm*89, *n* = 8; *emm*109, *n* = 1; *emm*122, *n* = 1). In these isolates the absence of SpeB probably resulted from changes other than the impairment of CovRS or RopB function, possibly due to mutations in one of the at least 19 other genes whose products have been shown to influence the production of an enzimatically active SpeB (Carroll and Musser, 2011). No *emm* type was associated with the absence of detectable SpeB activity and the only ST (ST382 representing 65% of all *emm*6 isolates) associated with a lack of SpeB activity (*p* = 0.004) presented no null alleles at any of the sequenced loci. However, isolates of this ST harbor multiple amino acid substitutions in both *covS* and *ropB* genes that could contribute to the speB-negative phenotype (Dataset 1).

## Discussion

Antimicrobial resistance among SSTI isolates was not significantly different from that found among contemporary iGAS and pharyngitis isolates, with the decreasing resistance to macrolides in SSTI mirroring declines in resistance previously described among isolates causing these other infections (Friães et al., 2013b; Silva-Costa et al., 2015).

The studied SSTI isolates presented a very high genetic diversity, which was higher than the diversity of iGAS isolates in the period of 2005–2009. Still, two lineages accounted for more than one third of all SSTI isolates, namely *emm*89 and *emm*1. The molecular epidemiology of SSTI GAS isolates varies considerably among the different published studies, but these often refer to distinct time periods than the one studied here and include different infection types, with some including only isolates from impetigo, while others, like the present study, include all SSTI isolates. In Finland, a clear dominance of *emm*77 (40%) was found among pus isolates from 2008, followed by *emm* types 1, 28, and 89 (13%) (Vähäkuopus et al., 2012). In Northern Spain, *emm*77 was the second more prevalent type (17%) among dermal infections during 2005–2011, but *emm*89 largely dominated (31%) (Tamayo et al., 2014). In Norway, skin isolates recovered between 2005 and 2006 were dominated by *emm* types 28, 12, and 87 (Kittang et al., 2008), and in Serbia *emm*58 was significantly associated with SSTI, comprising 12 of the 52 SSTI isolates recovered in 2001–2007 (Mijač et al., 2010). In Beijing, among 52 impetigo GAS isolated between 2005 and 2008, *emm*12 and *emm*1 were highly prevalent (54 and 37%, respectively) (Liang et al., 2005). In Japan, *emm*28 was the most prevalent (23%) among 53 abscess isolates during 2003–2006 (Wajima et al., 2008), while in Taiwan a high prevalence of *emm*11 and *emm*106 was reported among isolates from different types of SSTI (Chiang-Ni et al., 2011; Lin et al., 2011). A very high diversity was observed among isolates recovered from pyoderma in India and in the Aboriginal Australian communities, presenting *emm* types infrequently isolated in developed, temperate climate regions (McDonald et al., 2007; Kumar et al., 2012).

In our study, although most of the *emm* types were identified among both iGAS and SSTI isolates, there were clear differences in the overall *emm* and clonal distribution between the two infections. The *emm* type 1 associated to iGAS was also previously found to be overrepresented among iGAS relative to pharyngitis (Friães et al., 2012), confirming the enhanced capacity of this lineage to cause invasive disease. The *emm*1 isolates have remained a major cause of iGAS in Portugal from 2000 to 2009 (Friães et al., 2007, 2013b), in line with the worldwide dissemination of the M1T1 clone (O'Loughlin et al., 2007; Aziz and Kotb, 2008; Luca-Harari et al., 2009). The association between *emm*3 and iGAS is not unexpected, since *emm*3 has been reported as a major cause of invasive disease in Portugal and other countries, although the comparison with pharyngitis isolates did not identify this *emm* type (Beres et al., 2004; Luca-Harari et al., 2009; Friães et al., 2013b).

The association between *emm*89 and SSTI has been previously reported in Northern Spain, but with no information regarding the specific clades involved (Tamayo et al., 2014). In Finland, *emm*89 was not particularly associated with SSTI, although it was among the most frequent *emm* types in isolates recovered from pus (Vähäkuopus et al., 2012). However, the Finish study refers to 2008, prior to the emergence of *emm*89 clade 3 in Finland, at least among GAS isolated from blood (Latronico et al., 2016). In Portugal, only *emm*89 isolates lacking the *hasABC* locus, presumed to belong to the recently emerged clade 3 (Friães et al., 2015a; Zhu et al., 2015), were significantly associated with SSTI. This observation is in line with the previously reported increase in the prevalence of *emm*89 among SSTI in Portugal, but not in iGAS or pharyngitis, associated with the emergence of this acapsular clade (Friães et al., 2015a). In agreement, between 2005 and 2009 the increase of *emm*89 isolates lacking the capsule locus translated into a significant increase of *emm*89 isolates only among SSTI, but not among iGAS. Our data thus raises the possibility that the genome remodeling underlying the emergence of *emm*89 clade 3 may have led to a particular propensity to cause SSTI, although this clade also quickly replaced the previously circulating *emm*89 clades in all infection types (Friães et al., 2015a). This association with SSTI could be due to an increased capacity to colonize the skin, as well as to an improved ability to overcome the major host defense mechanisms present in skin and soft tissue or to produce cytotoxic effects at these sites. Increased transmissibility and persistence has been suggested for clade 3-associated strains based on enhanced capacity to adhere to uncoated plastic (Turner et al., 2015), while the increased expression of NADase and SLO by clade 3 has been associated with virulence in a mouse model of necrotizing fasciitis (Zhu et al., 2015). However, further investigation is needed to clarify the role of these phenotypes in the specific association of *emm*89 clade 3 with SSTI.

These associations between *emm* type and disease presentation were reflected on the different prevalence of the respective STs and SAg profiles between iGAS and SSTI, in agreement with the high congruence previously observed between these three typing methods (Carriço et al., 2006; Friães et al., 2013a). The observed association of *emm* clusters A-C3 and A-C5 with iGAS, as well as that of cluster E4 with SSTI also reflects the association of the respective *emm* types with the types of infection.

According to the predicted ability of the different M proteins to interact with host factors (Sanderson-Smith et al., 2014), invasive isolates would be associated with the ability to bind fibrinogen and albumin. The ability to bind fibrinogen was proposed as a mechanism to decrease complement deposition resulting from the activation of the classical pathway (Carlsson et al., 2005). A similar function is believed to be performed by C4BP (Carlsson et al., 2003), whose binding was associated with SSTI isolates due to the high prevalence of *emm* clusters E3 and E6 in this type of infection. The reasons why complement inhibition could be achieved through fibrinogen in iGAS and C4BP recruitment in SSTI remain elusive. Most *emm* clusters include proteins able to bind albumin, with the exception of cluster E4 (Sanderson-Smith et al., 2014). The association of cluster E4 with SSTI resulted in the overrepresentation of albumin binding among iGAS. Although albumin binding was shown to mask epitopes in the C-repeated region of the M protein (Sandin et al., 2006), deletion of this region did not impair virulence in a mouse intraperitoneal infection model (Waldemarsson et al., 2009), not providing clues as to why this could be important in the context of iGAS. The ability to bind to human IgA and IgG was associated with SSTI isolates. Immunoglobulin binding was shown to hinder opsonophagocytosis, even in the absence of a specific immune response (Carlsson et al., 2003). One can imagine such defense mechanism could be useful to bacteria in the context of both SSTI and iGAS and the reasons for the observed differences deserve further scrutiny. Only 5% of the SSTI isolates were predicted to bind plasminogen, although this was recently shown to facilitate keratinocyte invasion (Siemens et al., 2011) and could thus be beneficial in the context of SSTI.

The acquisition of mutations in the two-component regulatory system CovRS is regarded as an important mechanism promoting the transition to an invasive phenotype among GAS isolates. Current data suggests that upon infection with a wild type strain, *covRS* mutants arise at the focal infection site, from which mixed populations are recovered, and are subsequently selected for during transition to deeper, normally sterile sites (Sumby et al., 2006; Mayfield et al., 2014). In our previous work, we observed that null *covS* alleles were significantly overrepresented among iGAS (10%) relative to pharyngeal isolates (2%) (Friães et al., 2015b). Despite the low number of isolates carrying *covS* null alleles in all types of infection, the data reported here reveals an underrepresentation of *covS* null alleles in SSTI (3%) relative to iGAS isolates (*p* = 0.009), but no significant difference relative to pharyngeal isolates. Since our isolates were obtained from a single colony from each patient, there was a sampling of possibly mixed populations, which could introduce a bias or at least underestimate the ongoing selection of *covRS* impaired variants. Still we do not believe this compromises our conclusions since the colonies were randomly picked without particular care to select any of the variants. Our results therefore indicate that mutations impairing CovRS are a hallmark of iGAS isolates, although they still represent a minority of iGAS (Friães et al., 2015b). The lower prevalence of *covS* null alleles among pharyngeal and SSTI isolates supports the importance of a functional CovRS in the initial stages of non-invasive infection, both in skin and in the upper respiratory tract (Hollands et al., 2010; Alam et al., 2013). As reported for pharyngeal and invasive isolates (Friães et al., 2015b), *covRS* and *ropB* mutations occurred in SSTI isolates of diverse lineages and were not a particular characteristic of any specific clone. In contrast to *covS*, the proportion of *ropB* null alleles among SSTI isolates (4%) was similar to that found among both pharyngeal and invasive isolates (3%), indicating that mutations abrogating RopB activity occur in a low fraction of GAS isolates, regardless of the type of infection they cause.

The approach of considering as null alleles only those with premature stop codons can potentially underestimate the number of isolates with non-functional regulators, since some amino acid substitutions may also inactivate protein function. However, this conservative approach was based on the difficulty of confidently predicting the functional effect of missense mutations in *covRS* and *ropB*. Several studies have established an association between specific amino acid substitutions and an altered phenotype, such as reduced/absent SpeB or enhanced expression of hyaluronic acid capsule (mucoid colonies), SLO or NADase (Sumby et al., 2006; Hasegawa et al., 2010; Ikebe et al., 2010; Carroll et al., 2011; Olsen et al., 2012; Tatsuno et al., 2013). However, without functional studies comparing the phenotypes of the isolates with missense mutations and those of isogenic strains carrying the wild-type regulator genes, it is not possible to establish causality between the missense mutations and the complete loss of CovRS or RopB function. For a few mutations this correlation was further supported by the reversion of the phenotype after complementation with wild-type *covRS* genes (Engleberg et al., 2001; Masuno et al., 2014), but those mutations were not identified in our study. On the other hand, it has been shown that amino acid substitutions in CovS may result in a partial function loss (Tatsuno et al., 2013) and that the reduction in the kinase or phosphatase activity of CovS has different effects in the transcriptomes of strains from distinct lineages (Horstmann et al., 2015). Likewise, some *ropB* missense mutations that were identified in multiple strains were associated with different levels of SpeB expression and activity (Olsen et al., 2012).

The abrogation of SpeB activity is usually regarded as one of the most important features of CovRS mutants contributing to the invasive phenotype, since this abrogation spares multiple virulence factors involved in invasive disease pathogenesis and evasion of host immunity (Carroll and Musser, 2011). The proportion of isolates lacking SpeB among our SSTI collection (25%) was similar to that found previously among iGAS (24%) and higher, but not significantly, than that found among pharyngitis isolates in Portugal (16%, *p* = 0.081) (Friães et al., 2015b). Only one ST was associated with a lack of SpeB expression (ST382) and both this ST and the *emm* type it expresses (*emm*6) were evenly distributed in both SSTI and iGAS. These results suggest that, in addition to SpeB downregulation, the differential regulation of other virulence genes induced by impairment of CovRS is also critical for an enhanced invasive capacity of the isolates. In agreement with this suggestion, a mouse model of SSTI showed that *covRS* mutants produced larger necrotic regions than *speB* mutants (Engleberg et al., 2004).

A limitation of this study is the lack of information regarding the specific infection caused by the isolates, as well as the clinical evolution and outcome of each infection. SSTI include a wide range of superficial and deep infections which vary greatly in severity. It was therefore not possible to evaluate possible correlations between the clones identified in SSTI and the severity of the respective infections. This could bias the comparison between SSTI and iGAS, leading to a possible underestimation of the differences between the two types of infection. However, even in these conditions we still identified significant differences between the two subsets of isolates regarding both clonal composition and presence of null *covS* alleles, which are in agreement with our previous observations from the comparison between pharyngeal and iGAS isolates (Friães et al., 2012, 2015b).

GAS isolates causing SSTI in Portugal are a genetically diverse population. Still, the 30 valent M protein-based vaccine, which was shown to evoke cross-opsonic antibodies against non-vaccine serotypes (Dale et al., 2011), could potentially cover up to 95% of SSTI in Portugal. Although SSTI are the main primary foci associated with invasive disease (Lamagni et al., 2008), SSTI isolates, inasmuch as pharyngeal isolates (Friães et al., 2012), present a clearly different clonal composition from contemporary iGAS isolates. It is less clear how the differences in the presumed interaction with host proteins and exotoxin profiles brought about by those clonal differences could explain the distinct disease presentations. Despite the different prevalence of multiple *emm* types between SSTI and iGAS, within each *emm* type the same MLST defined lineages and the same SAg profiles could be found in both infection types. This indicates that any intra-*emm* type genetic differences between the two populations must be explored at a more detailed level, such as by whole-genome sequencing. Our current data therefore indicates that among the GAS clones causing infection in the Portuguese population, some have an increased capacity to invade deeper tissues and cause severe infections, while others seem to be particularly successful at establishing SSTI or pharyngeal infections. The proportion of SSTI isolates with no detectable SpeB activity was modest and similar to that found previously in Portugal among iGAS and pharyngitis isolates, indicating that the selective pressure to eliminate SpeB is also not a primary factor in SSTI. However, we confirmed an association between mutations abrogating CovRS function and iGAS, suggesting that increased expression of other virulence factors, such as the hyaluronic acid capsule, streptolysins or NADase, rather than SpeB downregulation, may be under selection in the context of iGAS.

## Data availability statement

The dataset generated and analyzed in this study can be found in FigShare [DOIs 10.6084/m9.figshare.6736313 and 10.6084/m9.figshare.7016663].

## Author contributions

CP performed the experiments. PGSSI collected data. AF, CP, and MR analyzed and interpreted the data. AF, CP, JM-C, and MR were involved in the conception and design of the study, as well as in drafting the manuscript. AF, CP, JM-C, MR, and PGSSI were involved in revising the paper critically for important intellectual content.

### Conflict of interest statement

JM-C has received research grants administered through his university and received honoraria for serving on the speakers bureaus of Pfizer and Merck Sharp, and Dohme. MR has received honoraria for serving on the speakers bureau of Pfizer and for consulting for GlaxoSmithKline and Merck Sharp and Dohme. The remaining authors declare that the research was conducted in the absence of any commercial or financial relationships that could be construed as a potential conflict of interest.
