# Supplementary material for: Streptococcus pyogenes Causing Skin and Soft Tissue Infections Are Enriched in the Recently Emerged emm89 Clade 3 and Are Not Associated With Abrogation of CovRS
Source: Front Microbiol. 2018 Oct 9;9:2372. doi: 10.3389/fmicb.2018.02372 (PMC6189468; doi:10.3389/fmicb.2018.02372)
Supplement: Supplementary file 1 [file Data_Sheet_1.PDF]

Supplementary Material

Streptococcus pyogenes Causing Skin and Soft Tissue Infections Are Enriched in the Recently Emerged *emm*89 Clade 3 and Are Not Associated with Abrogation of CovRS

Catarina Pato, José Melo-Cristino, Mario Ramirez, Ana Friães* and the Portuguese Group for the Study of Streptococcal Infections

*** Correspondence:** Ana Friães: afriaes@fm.ul.pt

Supplementary Table 1- Host protein binding profile prediction for each cluster (Sanderson-Smith *et al*. 2014 J Infect Dis 210:1325-38) and distribution of the isolates recovered from skin and soft tissue infections (SSTI) and invasive infections (iGAS) in Portugal during 2005-2009.

***emm* cluster**	**SSTI/iGAS [a]**	**Plasminogen**	**IgA**	**IgG**	**Fibrinogen**	**Albumin**	**C4BP**
E4	90/49	No	UC[b]	Yes	No	No	UC[b]
A-C3	46/70	No	No	Yes	Yes	Yes	No
E3	41/22	No	UC[b]	Yes	No	Yes	Yes
M6	20/21	No	No	No	No	UC[b]	UC[b]
A-C5	12/27	No	No	No	Yes	Yes	No
E1	20/15	No	Yes	Yes	No	Yes	Yes
E6	21/7	No	Yes	Yes	No	Yes	Yes
D4	16/9	Yes	UC[b]	No	No	UC[b]	No
A-C4	10/12	No	No	No	Yes	Yes	No
M5	4/6	No	No	No	Yes,	UC[b]	UC[b]
E2	4/2	No	No	Yes	No	Yes	No
M18	3/2	No	No	No	No	UC[b]	UC[b]
D2	3/0	No	No	No	No	UC[b]	UC[b]
M19	0/1	No	No	No	Yes	Yes	No
M74	1/0	No	No	No	Yes	UC[b]	UC[b]
M122	1/0	No	No	No	No	UC[b]	UC[b]
M179	0/1	No	No	No	Yes	UC[b]	UC[b]

[a] Results from iGAS *emm* typing were reported previously (Friães *et al*. 2007 J Clin Microbiol 45:2044-7; Friães *et al*. 2013 Eur J Clin Microbiol Infect Dis 32:115-25).
[b] When < 80% isolates were tested for each *emm* cluster the property is classified as uncertain (UC).

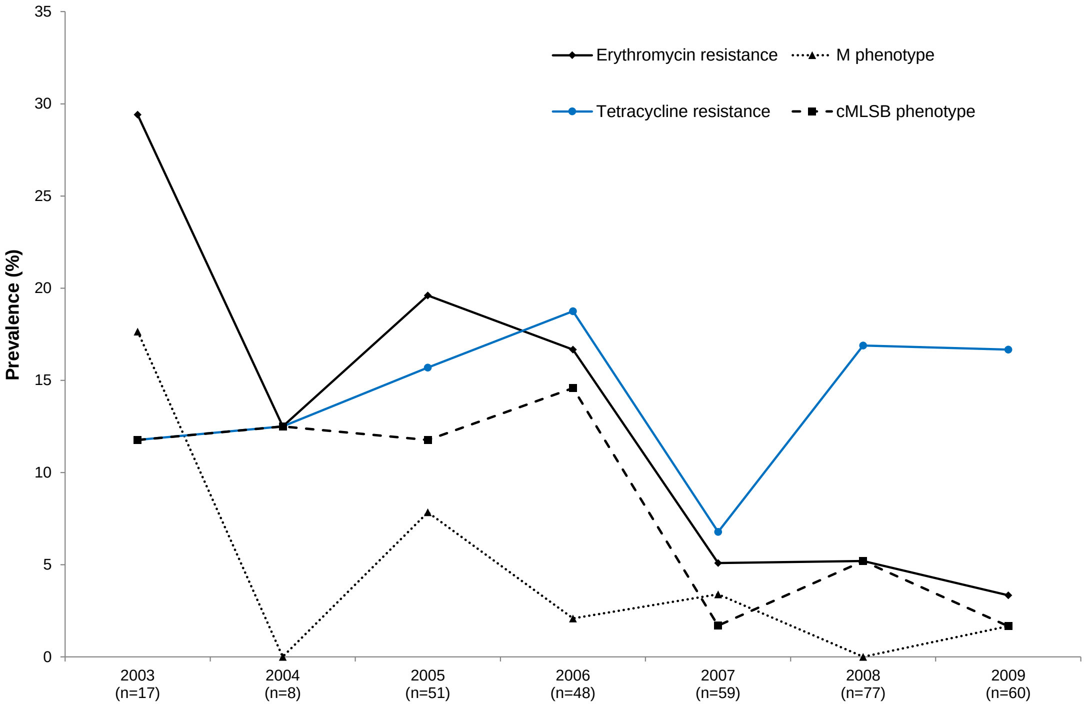

Supplementary Figure 1. Prevalence of tetracycline resistance, erythromycin resistance, and macrolide resistance phenotypes among GAS isolates recovered from skin and soft tissue infections (SSTI) in Portugal during 2003-2009. M, resistance to erythromycin and susceptibility to clindamycin; $cMLS_B$, constitutive resistance to erythromycin and clindamycin.

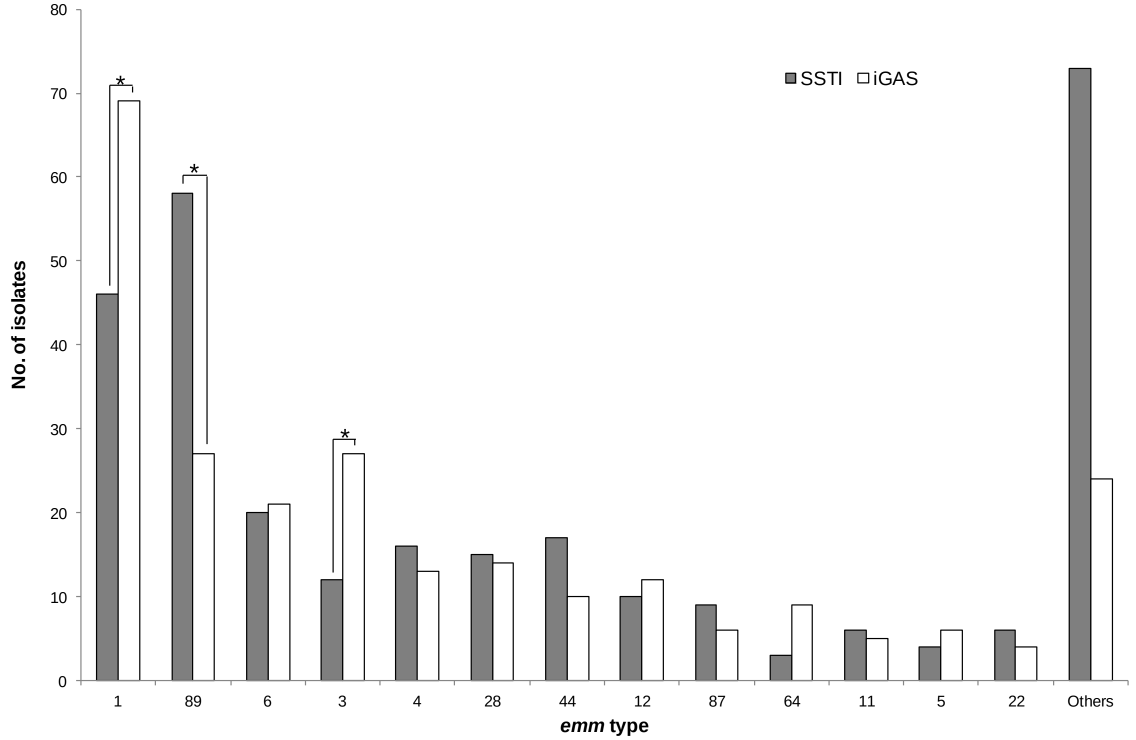

Supplementary Figure 2. Distribution of *emm* types among isolates recovered from skin and soft tissue infections (SSTI) and invasive infections (iGAS) in Portugal during 2005-2009. “Others” include *emm* types with a total of <10 isolates in both infection types [SSTI: *emm*2 (*n* = 8), *emm*48 (*n* = 7), *emm*75 (*n* = 5), *emm*58, *emm*78, and *emm*118 (each *n* = 4), *emm*9, *emm*18, *emm*53, and *emm*71 (each *n* = 3), *emm*70, *emm*76 and *emm*223 (each *n* = 2), *emm*33, *emm*43, *emm*50, *emm*65, *emm*74, *emm*77, *emm*80, *emm*81, *emm*82, *emm*83, *emm*90, *emm*93, *emm*94, *emm*102, *emm*103, *emm*109, *emm*122, *emm*127, *emm*147, *emm*167, *emm*168, *emm*209, and *emm*225 (each *n* = 1); iGAS: *emm*18, *emm*58, *emm*77, *emm*78, and *emm*103 (each *n* = 2), *emm*2, *emm*9 *emm*19, *emm*75, *emm*76, *emm*81, *emm*84, *emm*90, *emm*118, *emm*179, *emm*196, *emm*199, *emm*227, and stG1750 (each *n* = 1)].*$p \leq 0.01$.

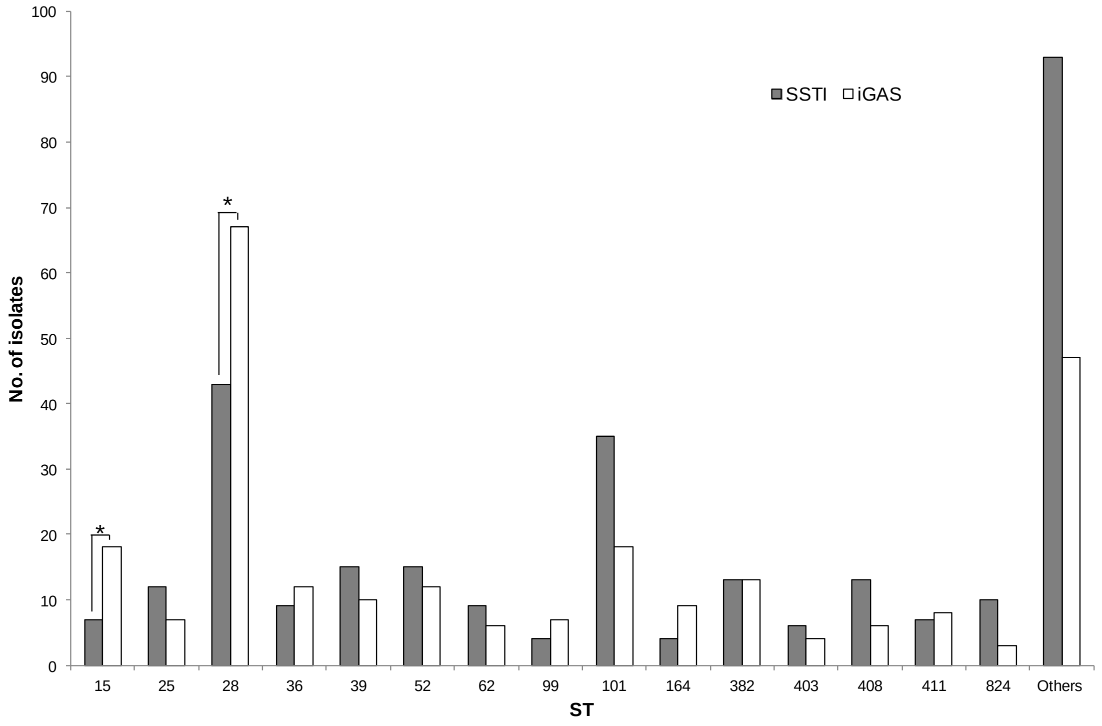

Supplementary Figure 3. Distribution of sequence types (ST) among skin and soft tissue infections (SSTI) and invasive infections (iGAS) in Portugal during 2005-2009. “Others” include STs with a total of <10 isolates in both infection types [SSTI: ST55 (*n* = 8), ST161 (*n* = 7), ST46 and ST315 (*n* = 5), ST75, ST130, ST150, ST253, and ST826 (each *n* = 3), ST3, ST24, ST50, ST178, ST340, ST555, ST565, ST642 and ST830 (each *n* = 2), ST2, ST5, ST10, ST38, ST60, ST63, ST89, ST120, ST166, ST167, ST184, ST200, ST341, ST389, ST409, ST429, ST467, ST569, ST573, ST643, ST701, ST718, ST754, ST819, ST820, ST822, ST825, ST827, ST828, ST829, ST831, ST833, ST834, ST835, and ST836 (each *n* = 1); iGAS: ST406 (*n* = 5), ST46 and ST315 (*n* = 4), ST643 (*n* = 3), ST63, ST201, ST409, ST410, ST555, and ST823 (each *n* = 2), ST50, ST55, ST75, ST95, ST150, ST178, ST184, ST258, ST402, ST458, ST562, ST618, ST619, ST769, ST771, ST816, ST818, ST821, ST832, and ST833 (each *n* = 1)]. *$p < 0.01$.

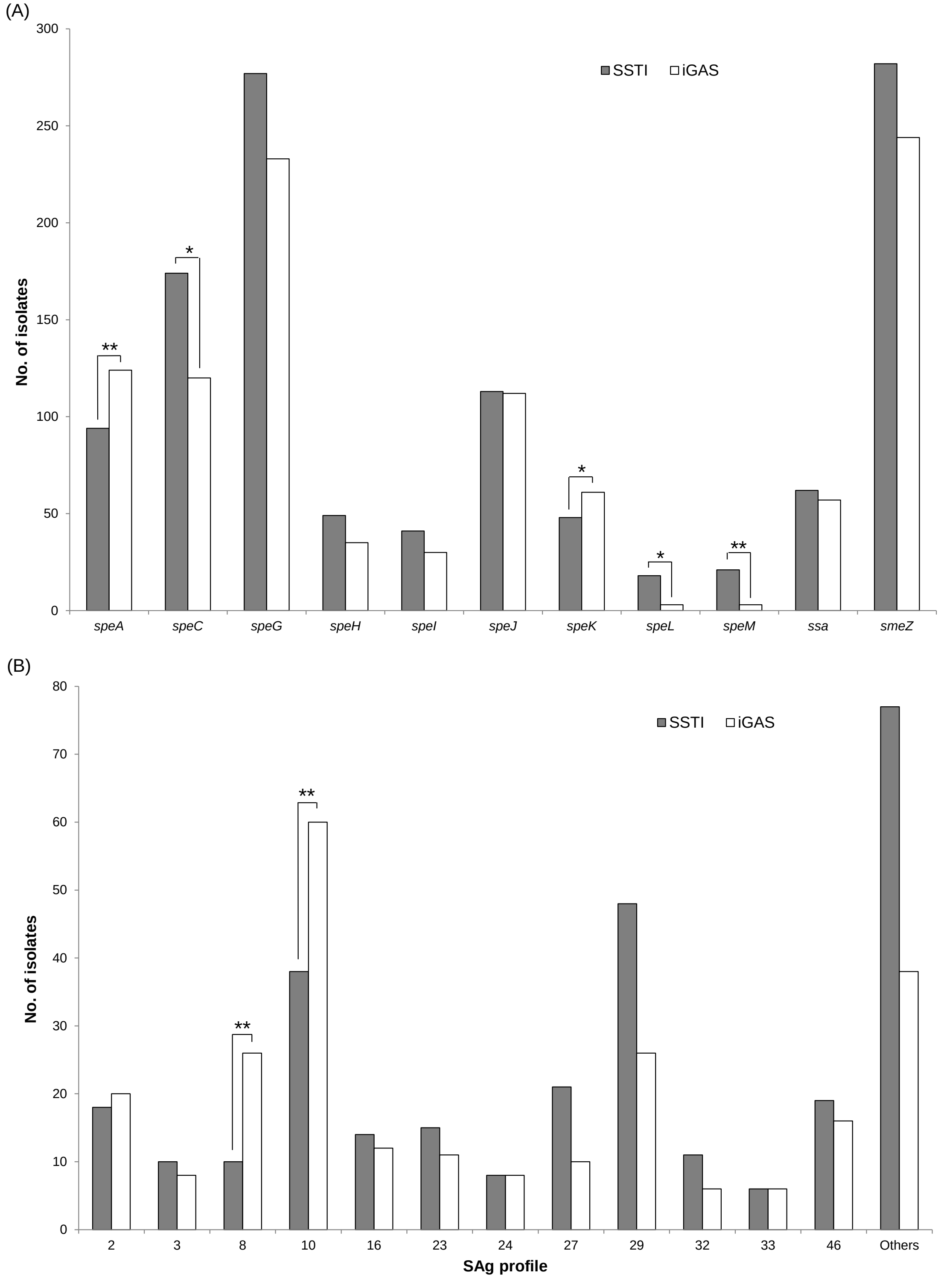

Supplementary Figure 4. Distribution of individual superantigen (SAg) genes (A) and SAg profiles (B) among isolates recovered from skin and soft tissue infections (SSTI) and invasive infections (iGAS) in Portugal during 2003-2009. “Others” include SAg profiles with a total of <10 isolates in both infection types [SSTI: SAg profiles 31 (*n* = 7), 5 and 44 (each *n* = 5), 20 and 40 (each *n* = 4), 4, 26, 38, 39, 56, and 66 (each *n* = 3), 11, 12, 21, 35, 47, 51, 53, and 60 (each *n* = 2), 9, 13, 15, 18, 19, 28, 41, 43, 45, 48, 52, 54, 64, 67, 68, 69, 70, and 71 (each *n* = 1); iGAS: SAg profiles 20 (*n* = 5), 5 and 44 (*n* = 4), 26 (*n* = 3), 17, 19, 30, 38, 52, 54, and 56 (each *n* = 2), 1, 12, 26, 31, 34, 45, 50, 51, 53, 55, and 57 (each *n* = 1)]. *$p < 0.05$ **$p < 0.01$.
